# Non-Isothermal Thermogravimetry of Selected Tropical Woods and Their Degradation under Fire Using Cone Calorimetry

**DOI:** 10.3390/polym13050708

**Published:** 2021-02-26

**Authors:** Linda Makovicka Osvaldova, Ivica Janigova, Jozef Rychly

**Affiliations:** 1 Department of Fire Engineering, Faculty of Security Engineering, University of Zilina, 01026 Zilina, Slovakia; 2 Polymer Institute, Slovak Academy of Sciences, 84541 Bratislava, Slovakia; ivica.janigova@savba.sk (I.J.); jozef.rychly@savba.sk (J.R.)

**Keywords:** tropical wood, non-isothermal thermogravimetry, deconvolution of thermogravimetry runs, cone calorimetry testing, heat-release rate

## Abstract

For selected tropical woods (Cumaru, Garapa, Ipe, Kempas, Merbau), a relationship was established between non-isothermal thermogravimetry runs and the wood weight loss under flame during cone calorimetry flammability testing. A correlation was found for the rate constants for decomposition of wood in air at 250 and 300 °C found from thermogravimetry and the total time of sample burning related to the initial mass. Non-isothermal thermogravimetry runs were assumed to be composed from 3 theoretical runs such as decomposition of wood into volatiles itself, oxidation of carbon residue, and the formation of ash. A fitting equation of three processes was proposed and the resulting theoretical lines match experimental lines.

## 1. Introduction

Wood is a natural, flammable material, and therefore fire-risk properties represent some obstacle for its applicability in constructions. The fire-technical properties of common woods such as spruce, oak, beech, etc. [1,2,3,4] used in different constructions and buildings have been given much attention, while less attention has been paid to tropical woods as they represent more complex systems [5,6]. Differences in fire behavior from that of the classical woods may be the result of the tropical woods’ morphology, differences in content of hemicelluloses, cellulose, and lignin, as well as the characteristics and amount of inorganic and organic substances of low molar mass.

Tropical and subtropical trees have been imported, processed, and sold in Central Europe since the end of the 1970s, desirable for their excellent physical and mechanical characteristics and innovative color finish in comparison with European native trees. The good stability and lifespan of some exotic tree species make them the ideal choice for decking balcony and swimming pool flooring, etc., especially in places with increased humidity and high probability of sudden weather changes [7]. Wood from tropical trees is used both in natural and modified forms, such as Thermwood [8,9]. However, as with any wood, tropical woods are a natural combustible material, and their fire properties are worth of further study. Less attention has been paid to tropical woods, regarding their potential fire-risk, than to the more typical woods such as e.g., spruce, oak, beech, etc. [10,11,12,13] used in different constructions and buildings. Tropical woods are more complex systems [1,4,5], leading to more difficulty in characterizing their fire properties. Comparison of the combustibility of woods of different origin can be complicated due to differences in density, and therefore different initial masses of samples at uniform dimensions; this disparity may affect the time to ignition. There are also the different admixtures of both organic and inorganic nature with the result that unambiguous ordering of combustibility may be more complex [14,15,16,17,18].

This contribution, a continuation of previous research [19] is an attempt to characterize the thermal stability of 5 tropical woods, three of South American origin (Cumaru, Garapa, Ipe) and two of Southeast Asian origin (Kempas and Merbau), by non-isothermal thermogravimetry in air and oxygen. Simultaneously, the accompanying changes in mass during burning were monitored using cone calorimetry. The burning was characterized by the classical parameters read from cone calorimetry such as MARHE (Maximum average rate of heat emission) and FIGRA (fire growth rate) as well as by the effective heat of combustion and consumption of oxygen.

However, it is worth noting that in addition to its combustion or mechanical resistance properties, the choice of a wood for a particular application must always be conditioned to the careful analysis of its sustainable character regarding its conditions of exploitation and availability of its species to long term. The goal of this research is not therefore to support the extreme deforestation and illegal logging of rainforests, but to do research on the types of trees which might be used in buildings, but to partially examine their response to fire. In the case of some tropical tree species (in natural form or modified), getting to know as much as possible about their fire properties (willingness to ignite and burn) may limit their use in practice, as they might not meet the fire regulations of some countries. By pinpointing these, we can regulate and limit their wider use.

## 2. Materials and Methods

### 2.1. Materials

The samples came from a Slovak supplier DLH SLOVAKIA s.r.o. in the form of boards without any surface treatment. Three were from the South American continent—Cumaru, Garapa, Ipe—and two from South-east Asia—Kempas and Merbau. Specification lists of respective woods are in papers [19,20].

Samples were selected carefully regarding their moisture content and density. The samples were selected from the cut boards such that samples’ density did not differ by more than ± 5% kg/m^3^. The content of water in the samples was measured gravimetrically and conditioned to constant weight.

The density of the samples (kg/m^3^) at 12% relative humidity was: Cumaru (1070) > Ipe (1050) > Kempas (880 > Merbau (830) > Garapa (790).

Samples of the dimensions 100 × 100 × 20 mm (±1 mm) were used in cone calorimetry testing. 4 parallel experiments were performed for each wood type.

**Figure 1:**
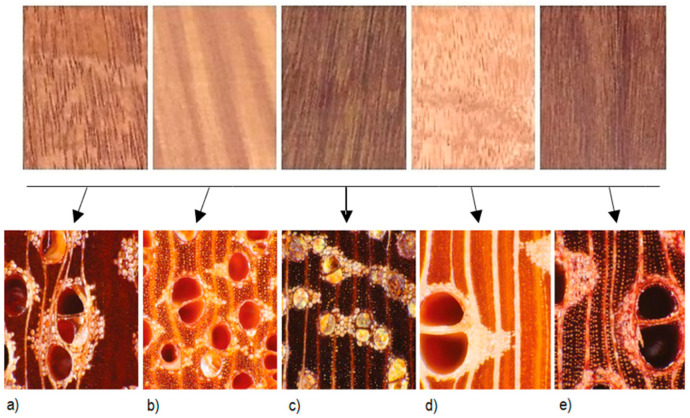
Microscopic structure (cross-section) of tropical tree species from listed as: (**a**) Cumaru, (**b**) Garapa, (**c**) Ipe, (**d**) Kempas, (**e**) Merbau [19].

Figure 1 shows the cross-section and microscopic structure of the selected tree species: (a) Cumaru, (b) Garapa, (c) Ipe, (d) Kempas, (e) Merbau.

### 2.2. Method

#### 2.2.1. Thermogravimetry

The wood board samples were reduced to sawdust; the mass used for the measurements was between 1–2 mg.

The change of the sample mass with increasing temperature was measured using a Mettler–Toledo TGA/SDTA 851^e^ instrument with a gas flow of 30 mL/min, in a temperature range from room temperature up to 550 °C and a heating rate of 10 °C/min. Temperature calibration was performed with Indium and aluminum standards. The repeatability of the experiments was excellent; the scatter between two parallel measurements in the temperature region of an active decomposition between 300–400 °C was lower than 1 °C. The average line from two parallel measurements was taken [21,22,23].

#### 2.2.2. Thermogravimetry Reaction Scheme

The formation of volatile degradation products from wood, as observed by non-isothermal thermogravimetry, is a complex process. Before the appearance of molecules capable of escaping the system, wood may undergo a sequence of reaction steps that are essentially based on cleavage of the weakest bonds in the macromolecules of cellulose, hemicellulose, and lignin. This results in smaller fragments which are both volatile and nonvolatile under instantaneous conditions. Simultaneously, depolymerization may occur, as well as crosslinking that may enormously complicate the process of the formation of volatiles [20,21,24].

**Scheme 1:**
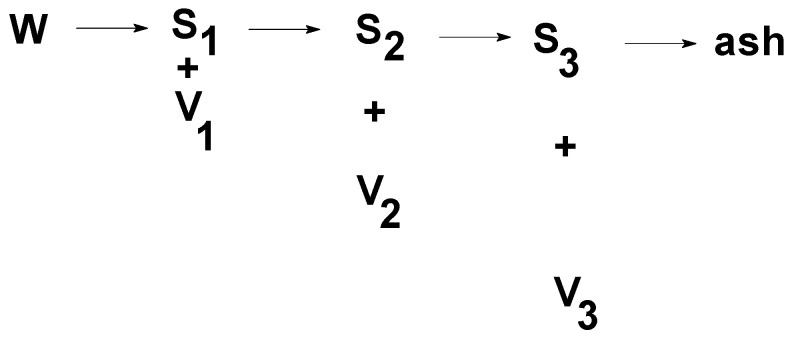
Wood decomposition as the sequence of component processes.

In this paper, we introduce Scheme 1 of wood W decomposition into volatiles V, where S are the products remaining on the reaction pan.

**Scheme 2:**
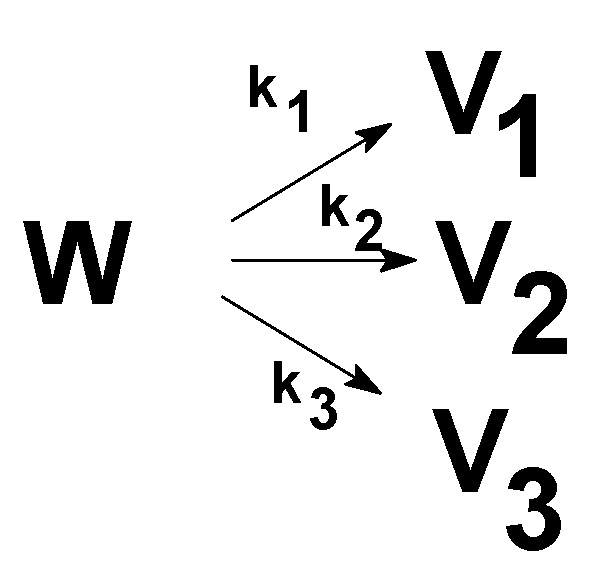
Simplified Scheme 1.

If the temperature program is appropriately chosen so that respective steps of Scheme 1 become well separated, then from the viewpoint of volatiles formed it may well be replaced by the Scheme 2 that we have used for the deconvolution of observed experimental lines [21].

The complex non-isothermal thermogravimetry curve may then be described as being composed of three independent processes each being described by the first-order scheme, i.e.,

(1) $$-\frac{dm}{dt}=km$$

In a non-isothermal mode,

$$-\frac{dm}{dT}\frac{dT}{dt}=Aexp\left(-\frac{E}{RT}\right)m$$

or alternatively,

(2) $$-\frac{dm}{m}=\frac{A}{\beta }\exp\left(-\frac{E}{RT}\right)dT$$

where *m*—actual mass, *T*—temperature in Kelvins, *R*—universal gas constant, *A* and *E*—pre-exponential factor and activation energy and is the linear heating rate.

$$\beta =\frac{dT}{dt}$$

After integration of this equation, we obtain:

(3) $$m=m_{0}\exp[-\frac{A}{\beta }\int_{T_{0}}^{T}\exp(-\frac{E}{RT})dT]$$

For the process composed of 3 temperature dependent components—“waves”, we have:

(4) $$m=m_{0}\overset{j}{\underset{i=1}{\sum }}\alpha_{i}\exp[-\frac{A_{i}}{\beta }\int_{T_{0}}^{T}\exp(-\frac{E_{i}}{RT})dT]$$

Provided that mass changes are expressed as a percentage of the initial mass, *m*_0_, the parameters $\alpha_{i},A_{i},E_{i}$, where *α*_i_ is a fraction of respective component process, may be found by a nonlinear regression analysis (Levenberg, Marquardt algorithm) applied to curves of the experimental mass m vs. temperature T, from the initial temperature *T*_0_ to a final temperature *T* of the experiment. The rate constant, *k_i_*, corresponding to a given temperature is expressed as $k_{i}=A_{i}\exp(-E_{i}/RT)$.

#### 2.2.3. Cone Calorimetry

The cone calorimeter used was product of Fire Testing Laboratory Ltd. UK. The peak release rate (PkHRR) from a cone calorimeter, a key factor in fire assessment, is measured by monitoring the oxygen consumption based on the difference of air flows between the cone entrance and the cone extraction pipe of the cone calorimeter system. Calculations can then be executed based on empirical knowledge of the combustion of macromolecules, namely that an average of 13.1 MJ of heat is released per one kilogram of consumed O_2_ [25]. The heat flow was set at 35 kW/m^2^.

Other readouts were ignition time, total burning time, weight loss, effective heat consumption (EHC), total O_2_ consumption.

Each sample of the given dimensions was placed into the steel frame in horizontal position. The only part of the sample exposed to heat was its surface, not its edges. To prevent the material from peeling and the degradation products from dripping, the sample was wrapped in aluminum foil on the bottom and the sides. The radiator, which has a shape of a truncated cone and provided a uniform source of radiation, was placed above the sample. The temperature was regulated using 3 thermocouples and a thermostat. Weight measurements were carried out using the load cell of a tensometer with a readability of 0.01 g. A 10 kV spark generator equipped with a safety shut-off mechanism was used to ignite the volatiles from the test specimens [26]. There was a steady flow of air of 24 l/h throughout the burning channel.

## 3. Results

### 3.1. Non-Isothermal Thermogravimetry

**Figure 2:**
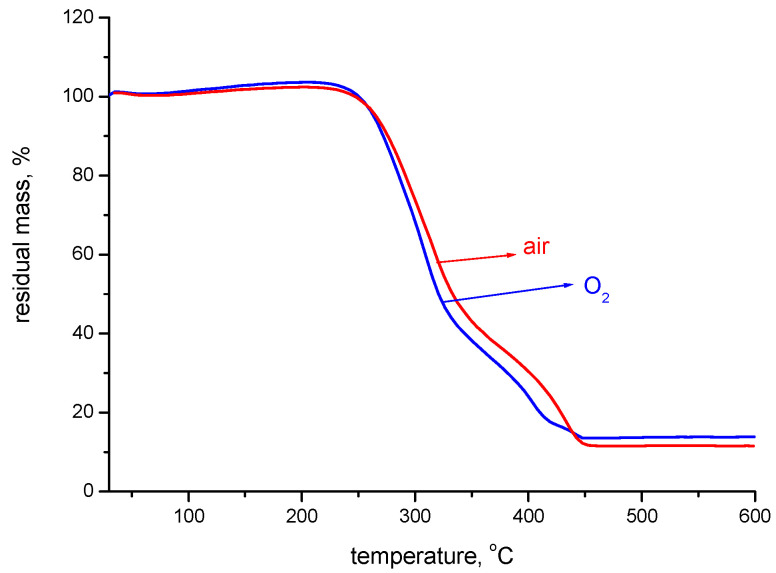
The average non-isothermal thermogravimetry–temperature runs (from 2 parallel experiments) in oxygen, and air for Cumaru wood at the rate of heating 10 °C/min.

Figure 2 demonstrates the effect of atmosphere (air and oxygen) on the mass loss of Cumaru wood when heated at a rate of 10 °C/min. The mass loss was precipitated by a slight increase in the mass, probably due to peroxidation. The main mass loss then is faster in oxygen than in air.

For deconvolution of experimental runs and for evaluation of corresponding pre-exponential factor and activation energy of the main fraction of volatiles formed from wood the function 1) and *j* = 3 was used.

**Figure 3:**
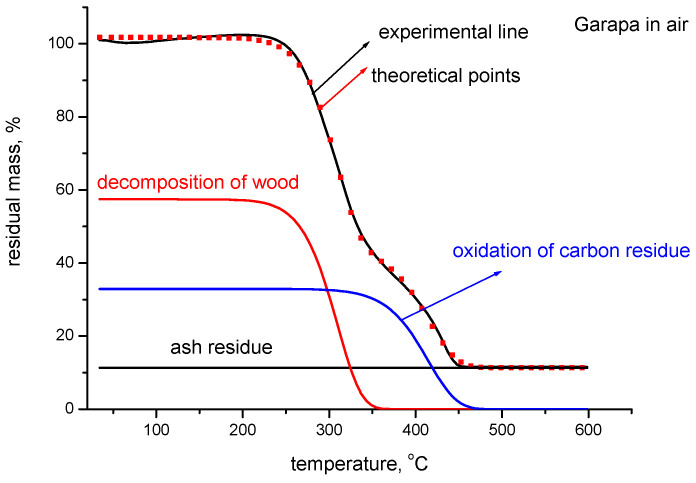
The deconvolution of non-isothermal thermogravimetry–temperature run into corresponding component processes with the use of Equation (1) for Garapa wood in air at the rate of heating of 10 °C/min. Points represent the theoretical run.

An example of such a deconvolution for Garapa samples in air is shown in Figure 3.

Table 1 gives the formal summary of parameters extracted from average experimental runs-mass of the sample vs. temperature according to Equation (4). The main component of the wood degradation is depicted in red. From this set of data, the rate constants of the wood decomposition were determined for 250 and 300 °C (Table 2). The decomposition of wood itself (red line), oxidation of carbon residue (blue line) and slight temperature changes of the ash (black line) can be seen.

### 3.2. Cone Calorimetry

**Figure 4:**
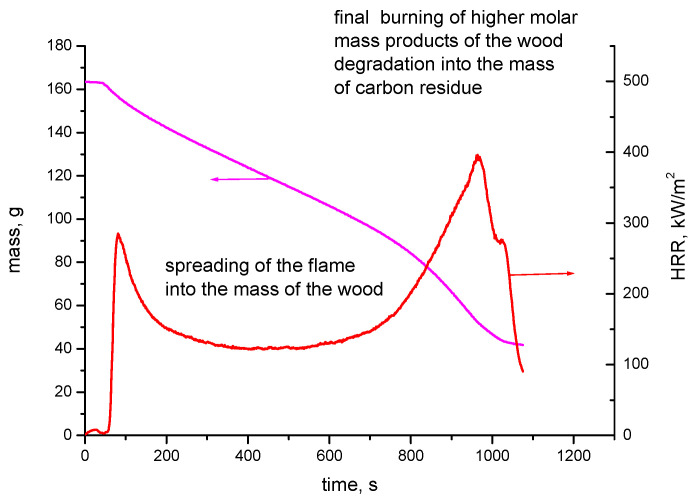
The average HRR–time and mass-loss runs (from 4 parallel experiments) at 35 kW/m^2^ for Cumaru.

The cone calorimetry runs were recorded by the averaging 4 parallel experiments with sample Cumaru (Figure 4); all important parameters for respective samples are in Table 3. In contribution [19] we stated that repeatability of respective experiments was high. Typical heat-release rate (HRR)–time runs involve a sharp increase of the heat released just after ignition. After that time, the flame is spread along the surface and, in its close proximity, the flame then penetrates through the layer of carbonaceous residue underneath.

It was assumed that the high-molar-mass products of the degradation of the wood’s degradation were pushed into the bottom of the carbonaceous layer by the heat front, and partially accumulate there. In the final stage of the wood burning, a second maximum appears, representing the burning of the accumulated products, followed by the extinction of the flame. The HRR–time runs are, in fact, a first derivative of mass-loss runs, as may be also seen on Figure 4. Table 3 presents the time of ignition, time of burning, HRR peak, MARHE, and FIGRA and other averaged parameters.

**Figure 5:**
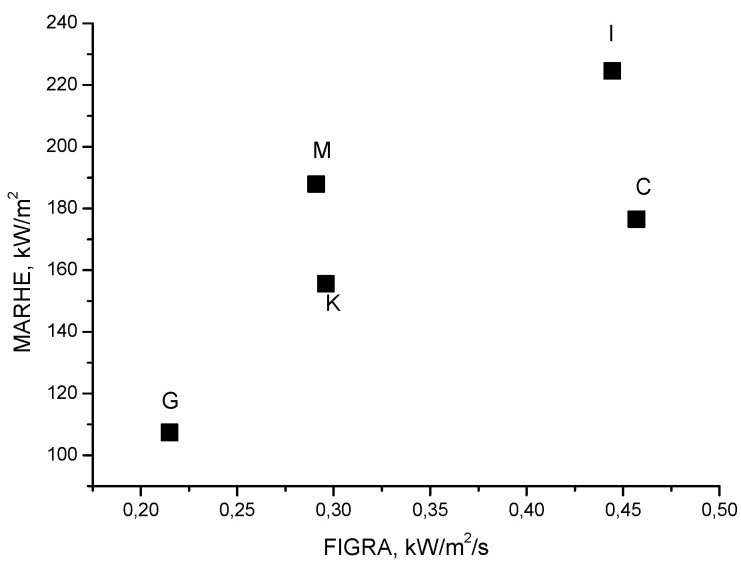
Correlation between MARHE and FIGRA parameters (average values) for respective samples of tropical woods: C—Cumaru, G—Garapa, I—Ipe, K—Kempas, M—Merbau.

When looking at Table 3, it is difficult to decide which of the five samples was the most flammable. However, when testing it by Bunsen burner shoved that Ipe and Merbau samples continued burning even when the burner was removed from direct contact with the sample, while Garapa, Kempas, and Cumaru extinguished. This alights with the values of MARHE parameters related to the second maximum, as well as with FIGRA (Figure 5). It is generally valid that the higher MARHE and/or FIGRA, the more flammable sample is.

**Figure 6:**
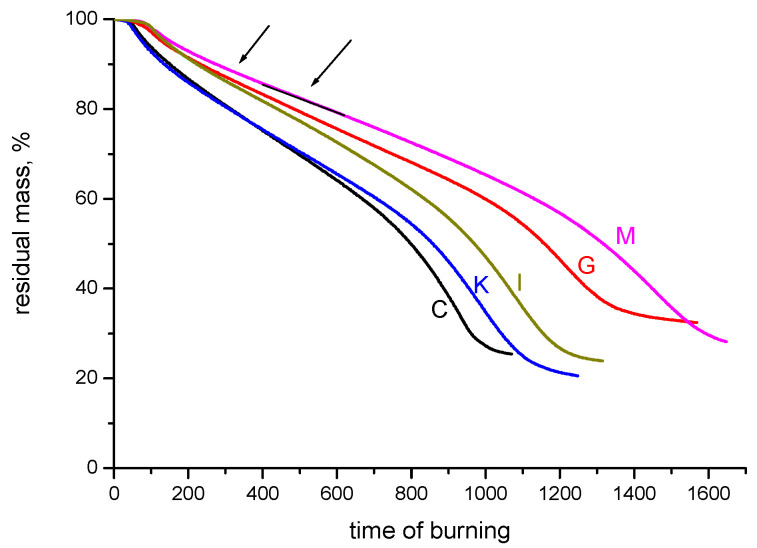
The average mass loss–time runs (from 4 parallel experiments) at 35 kW/m^2^ for C—Cumaru, G—Garapa, I—Ipe, K—Kempas, M—Merbau.

Figure 6 shows the average plots of mass loss related to 100% of the original mass during the burning of the woods. In the region of almost-linear decay, designated by arrows, quasi-isothermal conditions may be expected to occur on the surface of the burning material.

**Figure 7:**
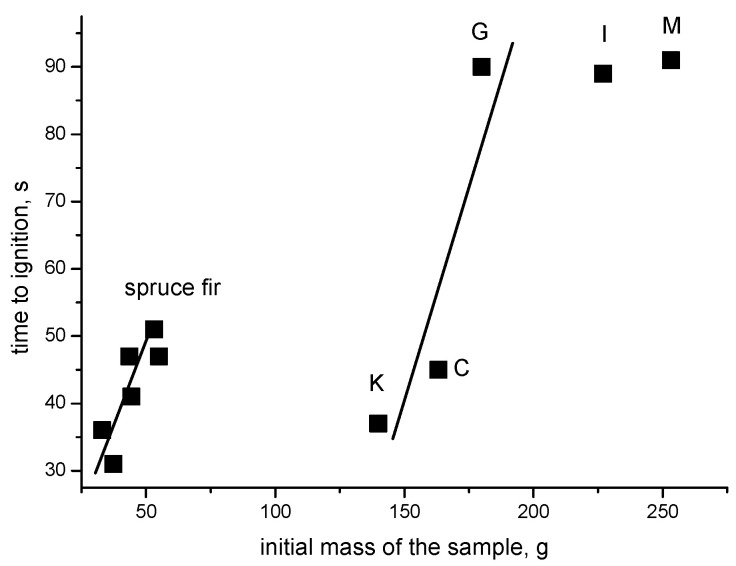
Time to ignition in dependence on initial mass. The cone radiancy was 35 kW/m^2^. The times of ignition of spruce were presented in the paper [19], G—Garapa, C—Cumaru, K—Kempas, I—Ipe and M—Merbau.

The density of the wood, and thus the initial mass at identical dimensions, is one of the factors determining the time to ignition (Figure 7). For samples of the same quality, (spruce) this is quite evident, while tropical trees differing also in structure and the amount of other additional compounds show larger scatter of respective experiments. Kempas, Cumaru, and Garapa approximately follow the straight line, but Ipe and Merbau decline. Ipe and Merbau samples have lower time to ignition than expected from the linear plot of Figure 7, probably because they are more ignitable than other woods.

**Figure 8:**
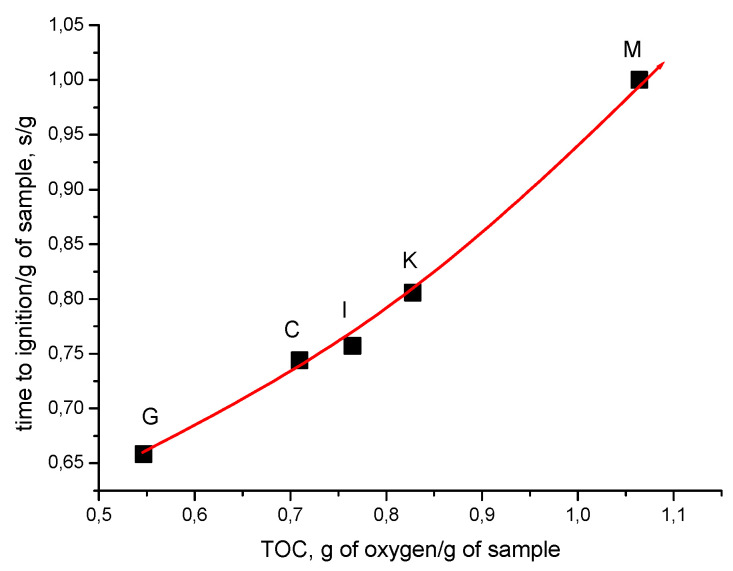
Correlation between time to ignition and the total oxygen consumed (TOC) related both to the initial mass of the sample for C—Cumaru, G—Garapa, I—Ipe, K—Kempas, M—Merbau.

**Figure 9:**
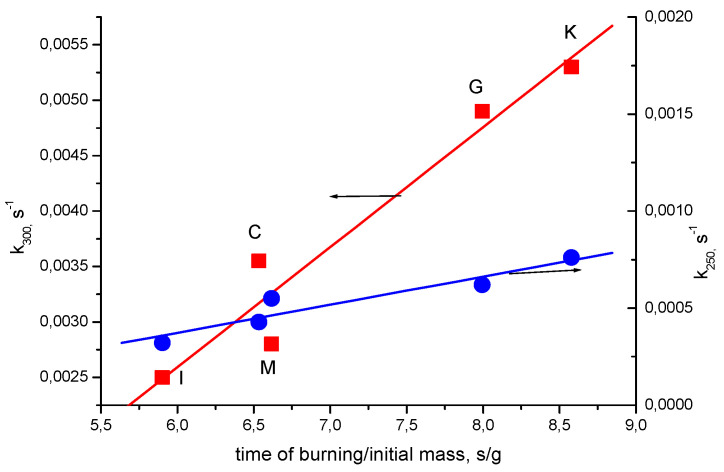
The rate constants at 250 and 300 °C determined from non-isothermal thermogravimetry vs. the total time of burning related to the initial mass of wood C—Cumaru, G—Garapa, I—Ipe, K—Kempas, M—Merbau.

Time to ignition appears also to be related to the total amount of oxygen consumed in burning (Figure 8), as if the ignition requires more oxygen to successfully initiate the burning. A quite convincing demonstration of a possible link between thermogravimetry runs and cone calorimetry burning may be seen in Figure 9, where rate constants from non-isothermal thermogravimetry determined for 250 and °C correlate quite well with the total time of burning related to the initial mass of samples.

**Figure 10:**
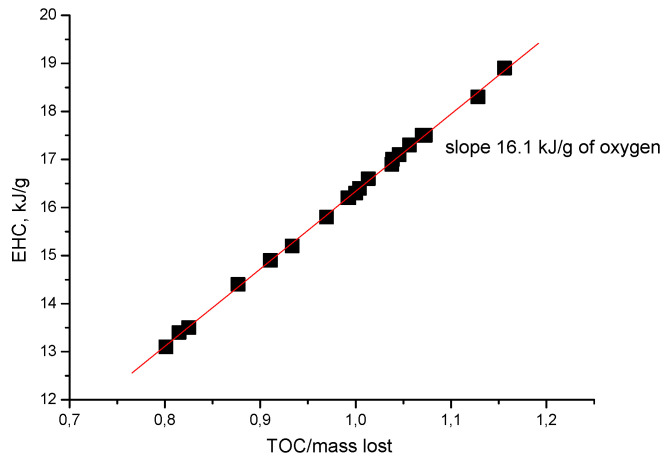
Universal correlation between EHC and total oxygen consumed related to unity of mass lost during burning (all parallel experiments involved).

Figure 10 shows the relationship between EHC and total oxygen consumed (TOC) divided by the mass lost during burning. The straight line produced in both cases of tropical woods, as well as spruce fir, has the shape: EHC = 1.008 + 16.1 × TOC/mass lost where the slope about 16 kJ/g is higher than parameter 13.1 kJ/g of consumed oxygen implemented in cone calorimeter. It may be of interest that the above correlation is also valid for spruce samples that were thinner than tropical woods [19].

## 4. Discussion

A correlation has been found for the rate constants for the decomposition of selected tropical woods in air at 250 and 300 °C, and the total time of sample burning related to the initial mass. The sequence of rate constants of wood degradation was as follows: Kempas > Garapa > Merbau > Cumaru > Ipe.

Decomposition of wood in thermogravimetry works with small initial mass of the sample, and thus with the direct release of volatile decomposition products into gaseous phase. However, in a cone calorimetry burning with much higher initial mass, the back effect of the heat from cone heater on degradation products formed during burning may keep them beneath the upper carbonaceous layer [27,28,29,30,31,32].

As expected, the time to ignition was affected by the initial mass of sample. This was valid for Cumaru, Ipe, and Kempas while Garapa and Merbau decline. This is probably due the fact that the latter two woods give the decomposition phenomenon composed formally from two processes [19,32,33,34,35,36].

The sequence of MARHE parameter related to the second maximum of the heat-release rate–time cone calorimetry runs is different from the sequence obtained from thermogravimetry. The order of flammability of examined woods is: Ipe (238) > Merbau (188) > Cumaru (176) > Kempas (155) > Garapa (107), given in kW/m^2^. This sequence correlates quite well with FIGRA parameters. At the same time, this may describe the relative tendency of the burning wood to retain some less volatile degradation products below the upper carbonaceous layer.

A linear correlation between EHC and TOC related to a mass lost during burning has been confirmed. The slope is however higher (about 16 kJ/g of consumed oxygen) than that implemented into cone calorimeter (13.1 kJ/g of consumed oxygen), which indicates that the stoichiometry of burning involves a significant portion of products such as carbon in the form of solid residue and soot, as well as carbon monoxide.

## 5. Conclusions

The degradation of tropical woods was evaluated in air and oxygen at a heating rate of 10 °C/min, while cone calorimetry tests were performed in air with a cone radiance of 35 kW/m^2^. The experimental conditions of decomposition of wood using thermogravimetry mean that considerably lower initial masses of a sample are used and the direct release of volatile decomposition products into the gaseous phase occurs. The sequence of the rate constants of wood degradation was as follows: Kempas > Garapa > Merbau > Cumaru > Ipe. The same decaying sequence was found for flammability of samples expressed as a total time of their burning related to the initial mass.

The larger initial mass of the samples in cone calorimetry burning created the conditions for the back effect of the heat from the cone heater contributing temporary retention of higher-molar-mass degradation products burning beneath the upper carbonaceous layer, which is demonstrated by the appearance of the second maximum on curves of heat-release rate vs. time. This maximum is brought about by their subsequent release.

## Figures and Tables

**Table 1:** Set of parameters from deconvolution of non-isothermal thermogravimetry runs (Equation (4)) for samples of tropical woods measured in air and oxygen (the rate of heating of 10 °C/min.) (α_i_ is the fraction ascribed to the respective component of the whole degradation process, i = 1,2 and 3).

Sample	Gas	α_1_	A_1_/β, (°C)^−1^	E_1_, kJ/mol	α_2_	A_2_/β, (°C)^−1^	E_2_, kJ/mol	α_3_	A_3_/β, (°C)^−1^	E_3_, kJ/mol
Cumaru	air	0.460	2.2 × 10^5^	97.4	0.465	8.6 × 10^7^	106.1	0.060	7.2 × 10^−6^	26.9
Garapa	air	0.110	28.2	166.3	0.574	6.6 × 10^7^	103.3	0.328	5.1 × 10^7^	121.5
Ipe	air	0.311	1.1 × 10^8^	133.4	0.602	2.2 × 10^7^	101.3	0.110	2.1 × 10^−8^	21.1
Kempas	air	0.302	6.7 × 10^3^	78.6	0.590	2.2 × 10^7^	97.6	0.124	3.2 × 10^−7^	37.0
Merbau	air	0.329	2.3 × 10^6^	113.5	0.604	3.6 × 10^5^	81.0	0.076	8.4 × 10^−7^	32.7
Cumaru	oxygen	0.438	8.2 × 10^4^	88.8	0.485	1.2 × 10^9^	116.6	0.081	4.0 × 10^−7^	31.3
Garapa	oxygen	0.136	19.6	160.3	0.554	2.6 × 10^9^	118.4	0.334	4.0 × 10^5^	41.2
Ipe	oxygen	0.337	0.095	104.5	0.572	5.6 × 10^8^	113.8	0.095	8.9 × 10^−4^	0.004
Kempas	oxygen	0.341	3.2 × 10^3^	71.4	0.596	4.5 × 10^7^	98.8	0.080	5.2 × 10^−7^	40.4
Merbau	oxygen	0.359	1.9 × 10^5^	95.2	0.599	5.8 × 10^6^	92.2	0.040	1.4 × 10^−8^	12.1

**Table 2:** Set of parameters extracted from deconvolution of non-isothermal thermogravimetry runs (the Equation (4), Table 1) for samples of tropical woods measured in air and oxygen decomposed with the rate of heating of 10 °C/min. The above set of data is related to second component process (the red data) which was used for determination of the rate constants of second component of wood mass loss at 250 and 300 °C.

Sample	Gas	α_2_	A_2_/β, (°C)^−1^	E_2_, kJ/mol	k_2_300 × 10^3^, s^−1^	k_2_250 × 10^4^, s^−1^
Cumaru	air	0.465	8.6 × 10^7^	106.1	3.6	4.3
Garapa	air	0.574	6.6 × 10^7^	103.3	4.9	6.2
Ipe	air	0.602	2.2 × 10^7^	101.3	2.5	3.3
Kempas	air	0.590	2.2 × 10^7^	97.6	5.3	7.6
Merbau	air	0.604	3.6 × 10^5^	81.0	2.8	5.5
Cumaru	oxygen	0.485	1.2 × 10^9^	116.6	5.5	5.4
Garapa	oxygen	0.554	2.6 × 10^9^	118.4	8.3	7.8
Ipe	oxygen	0.572	5.6 × 10^8^	113.8	4.6	4.8
Kempas	oxygen	0.596	4.5 × 10^7^	98.8	8.5	11.9
Merbau	oxygen	0.599	5.8 × 10^6^	92.2	4.3	6.9

**Table 3:** Parameters read from cone calorimetry measurements of tropical wood samples C (Cumaru), G (Garapa), K (Kempas), M (Merbau) and I (Ipe). Average from 4 parallel experiments.

Sample Average Line from 4 Parallel Runs	m Initial (g)	m_lost_ (g)	Time to Ignition/Time of Burning (s)	EHC (MJ/kg)	Peak HRR (kW/m^2^)	TOC (g)	MARHE (kW/m^2^)	FIGRA kW/m^2^/s
Cumaru	163.3	121.5	45 ± 4/1067 ± 30	15.6	457.2 ± 42.3	115.9	176.5 ± 5.1	0.457
Garapa	180.0	118.5	90 ± 10/1439 ± 49	13.6	275.0 ± 28.2	98.4	107.4 ± 6.3	0.215
Kempas	139.9	112.7	37 ± 2/1200 ± 34	17.1	307.8 ± 9.8	115.8	155.6 ± 7.4	0.296
Merbau	253.3	183.0	91 ± 16/1676 ± 50	17.4	438.4 ± 32,8	194.8	187.9 ± 8.4	0.291
Ipe	227.0	171.9	89 ± 5/1340 ± 64	16.9	500.9 ± 17.9	177.6	224.6 ± 8.6	0.444

Where m_lost_—total loss of mass during burning; time to ignition/time of burning–time to ignition is time interval from insertion of sample bellow cone heater to ignition (piloted ignition by spark)—time of burning is time of flame burning of sample until self-extinction; EHC—effective heat of combustion, Peak HRR—maximum heat-release rate corresponding to the second maximum of record; TOC—total oxygen consumed during combustion; MARHE—a maximum value of ARHE = (∫HRR*t* dt)/(t₁), integral in nominator is from time of ignition to t_1_—time of burning; FIGRA—the ratio of the peak HRR/time corresponding to this maximum.

## Data Availability

Data sharing not applicable.
